# Ultrasound Findings in Symptomatic HADD of the Hand and Wrist. A Case Series of 37 Patients

**DOI:** 10.1002/jcu.70312

**Published:** 2026-07-13

**Authors:** Marco Becciolini, Riccardo Picasso, Antonio Corvino, Federico Zaottini

**Affiliations:** ^1^ Misericordia di Pistoia Pistoia Italy; ^2^ Scuola Siumb di Ecografia Muscolo‐Scheletrica Pisa Italy; ^3^ Division of Radiology IRCCS Azienda Ospedaliera Metropolitana, Ospedale Policlinico San Martino Genova Italy; ^4^ Medical, Movement and Wellbeing Sciences Department University of Naples “Parthenope” Naples Italy; ^5^ Department of Health Sciences (DISSAL) University of Genova Genova Italy

**Keywords:** crystal arthropathies, joint capsule, ligaments, tendinopathy, ultrasonography

## Abstract

**Purpose:**

To present a series of 37 cases diagnosed with ultrasound (US) of symptomatic hydroxyapatite deposition disease (HADD) involving the wrist and hand.

**Materials and Methods:**

Reports from two institutions were searched using the keywords “HADD hand/wrist” or “calcium deposit hand/wrist”. The US examinations were performed with a similar standardized approach, focusing on the region of pain. B‐mode and Doppler sonograms were acquired. Each author reviewed the cases to ensure that only cases consistent with HADD were included.

**Results:**

A total of 37 patients were identified, mostly women (81% of the cases). The mean age was 50 years old. Calcifications usually showed resorptive stage features (type III). Follow‐up clinical data were available in 16 patients, mostly with at least a partial resolution of symptoms.

**Conclusion:**

US is a useful imaging technique to diagnose HADD of the hand and wrist, especially in the correct clinical context (e.g., acute non‐traumatic symptoms in women, with progressive improvement in pain). Conventional radiography may help to confirm the calcific deposit. Advanced imaging modalities are usually not required, but may be considered in selected cases. Follow‐up to confirm an improvement over time is suggested.

## Graphical abstract

Ultrasound enables accurate diagnosis of hydroxyapatite deposition disease of the hand and wrist in patients with acute, non‐traumatic pain, typically in middle‐aged women. Characteristic calcifications, often in the resorptive phase, support diagnosis, while radiography may confirm findings without the need for advanced imaging.

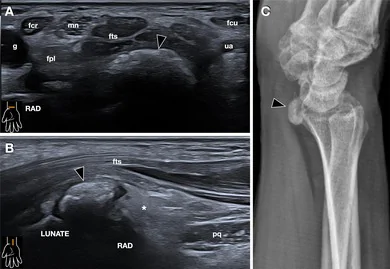

**Abbreviations**
BCP1: basic calcium phosphate
CMCj: carpometacarpal joint
CPPD: calcium pyrophosphate deposition disease
CTS: carpal tunnel syndrome
FCU: flexor carpi ulnaris
HADD: hydroxyapatite deposition disease
MCPj: metacarpal joint

## 1 Introduction

Hydroxyapatite deposition disease (HADD) is a common disorder that typically involves the tendons of the rotator cuff (Uhthoff et al. 1976; Siegal et al. 2009; Bureau 2013; Merolla et al. 2016; Ricci et al. 2022). Calcium deposits, also referred to as basic calcium phosphate (BCP) deposits, may involve anatomical sites other than the shoulder, including the hip, elbow, wrist, and knee. Although these calcific deposits are typically found in tendons, other tissues may be affected, such as ligaments, bursae, and capsules. HADD can be asymptomatic, particularly in the early phases of the calcification (formative and resting stages) (Omoumi et al. 2016). It occurs more commonly in women, with a mean age at presentation of 45 years (Moradi et al. 2014). Symptoms are usually related to the so‐called resorption phase of the calcium deposit, when they fragment and may migrate into the contiguous tissues (Bianchi and Becciolini 2019). Despite the acute pain that hampers daily activity and impairs sleep quality during the symptomatic phases, HADD is generally a benign self‐limiting condition, with symptoms decreasing and potentially resolving in 3–4 weeks (Beckmann 2016). The ultrasound (US) findings of HADD vary depending on the aforementioned stages of the calcification (Bianchi and Becciolini 2019). Although well described by Carroll already in 1955, HADD involvement is usually not considered in the differential diagnosis of hand/wrist non‐traumatic pain (Carroll et al. 1955). A possible term to describe finger involvement of HADD is “ Philadelphia finger” (Tamborrini et al. 2023).

This article aims to report the largest series of patients with an ultrasound diagnosis of symptomatic HADD of the hand and wrist, focusing on the relationship between clinical and imaging features, as well as on lesion location and the anatomical structures involved.

## 2 Materials and Methods

The institutional review board approved this retrospective study, and informed consent was obtained from all of the patients.

The US reports from two different institutions from January 2020 to December 2025 were searched using the words “HADD hand/wrist,” or “calcium deposit hand/wrist.” Static images and video clips were retrieved from the PACS system and reviewed along with the examination reports.

All patients presenting with acute painful symptoms within the previous 30 days in the area of the detected calcification and/or with chronic pain in the same region and a recent (within 1 month) acute exacerbation were included. Patients were screened and excluded if they had a history of gout or imaging signs of chondrocalcinosis. When clinical information could not be retrieved from the archives of the participating institutions, patients were contacted by telephone. Three clinicians at two sites (M.B., R.P., F.Z.) performed the US examinations, each with more than 10 years of experience in musculoskeletal US. High‐end machines, GE LOGIQ E10 at the first site and Canon i800 at the second, were employed, with a linear transducer with a large footprint (around 50 mm, GE ML6‐15 and ML4‐20; Canon i18LX5 and i24LX8) and a hockey stick smaller probe (footprint around 25 mm) and higher frequency (more than 18 MHz, GE L6‐24, Canon i22LH8) were used.

The US examination protocols for the examination of the patients were similar across both institutions. In particular, a systematic approach for the evaluation of the wrist/hand was adopted, focusing on the region of pain as reported by the patient (Picasso et al. 2023; Bianchi and Martinoli 2007; Bianchi et al. 2020; Klauser 2012; Lee and Healy 2005; Flores et al. 2023; Gitto and Draghi 2016).

Sonograms in real‐time were used to differentiate the calcific deposit from tendons, because calcifications remain hyperechoic even when changing the US beam direction, whereas tendons suffer from anisotropy and changes from hyper‐ to hypo‐echoic due to their fibrillar structure (Bianchi and Becciolini 2019). Care was taken to depict edematous changes of the adipose soft tissues near the calcific deposit, appearing as a hyperechoic halo. Color and/or Power Doppler imaging was used when a calcific deposit was documented in the B‐mode image to depict hyperemia. US‐guided pressure was useful to confirm the location of pain.

The maximal extension of the calcification was measured in mm, and the location of the deposit reported.

Additional findings (e.g., intraosseous migration of the calcification or synovitis) were also reported.

Each author independently reviewed the images, and only cases unanimously considered consistent with HADD by all three authors were included. We excluded cases that suggested other crystal deposition diseases, such as gout and chondrocalcinosis.

Correlation with other imaging modalities was available in some patients, as well as follow‐up data, including clinical data obtained at phone calls or at US re‐examinations.

For all of the patients, we also recorded the age, sex, and the clinical presentation.

## 3 Results

We identified 37 patients with an acute HADD of the hand/wrist.

The data are summarized in Table 1 and Figure 1.

**TABLE 1:** Demographic characteristics of the patients.

Pt	Sex	Age	Size	Location	Clinical presentation	Calc. Type	Edem Ch.	Dopp.	Other imaging	Associate findings	Follow‐up
1	F	73	47 mm	Volar wrist. Flexor tendons	Acute wrist pain 5 days before. CTS	Type III	Yes	Yes	X‐ray	Flexor synovitis	Phone. improvement 1 month
2	F	53	18 mm	Volar wrist. Ligament‐Capsule	Acute wrist pain 3 days before. CTS	Type III	Yes	Yes	X‐ray, MRI, CT	Intraosseus migration	US. Improvement 2 months
3	F	41	18 mm	Volar wrist. Ligament‐Capsule	Acute wrist pain 1 week, CTS	Type III	Yes	Yes	No	FCU deposit	US. Improvement 1 week
4	F	32	8.5 mm	Volar wrist. Ligament‐Capsule	Chronic wrist pain, with exacerbation 1 week before. mild CTS	Type III	No	Yes	No		No
5	F	56	14 mm	Volar wrist. Ligament‐Capsule	Chronic wrist pain, with recent exacerbation. CTS	Type I	No	No	No	CMCj oa	No
6	F	72	25 mm	Volar wrist. Ligament‐Capsule	Persistent CTS after surgery (pain recently exacerbated)	Type III	Yes	Yes	No	Flexor synovitis	No
7	F	65	22 mm	Volar wrist. Ligament‐Capsule	Acute wrist pain 15 days after CTS	Type III	Yes	Yes	No	Flexor synovitis	US. mild improvement 1 month
8	F	52	13 mm	Volar wrist. Ligament‐Capsule	Acute atraumatic volar wrist pain 10 days before. Symptoms improved with NSAID	Type II	No	Yes	No		No
9	F	45	8 mm	Volar wrist. Ligament‐Capsule	Acute pain 1 month before. Already improved at US	Type III	No	Yes	No		No
10	F	56	11 mm	Volar wrist. Ligament‐Capsule	Chronic wrist pain, recently exacerbated. CTS	Mixed. Mostly Type I, in part III	Yes	Yes	No	Flexor synovitis due to tendon sheath migration	No
11	F	59	15 mm	Volar wrist. Ligament‐Capsule	Acute volar wrist pain. CTS	Mixed. Mostly Type III, in part II	Yes	Yes	X‐ray	Volar ganglion cyst	No
12	M	45	12 mm	Volar wrist. Ligament‐Capsule	Acute volar wrist pain 2 days after minor trauma	Type III	Yes	Yes	X‐ray		Improvement with US‐guided injection. No follow‐up
13	M	45	5 mm	FCU	Chronic pain ulnar side wrist pain, recently exacerbated, mild dysesthesia 4–5th finger.	Type III	No	No	No	UN irritation	No
14	M	30	11 mm	FCU	Acute ulnar side wrist pain 5 days before. Slight improvement	Type III	Yes	Yes	No	Bursitis	Phone call 1 month. Resolved
15	F	65	6 mm	FCU	Pain ulnar side wrist since 1 month, partial regression at US examination	Type III	No	No	No	Bursitis	No
16	F	49	6 mm	FCU	Subacute pain ulnar side wrist	Type III	No	Yes	No	Intraosseus migration; PTj synovitis	No
17	F	55	7 mm	1st compartment retinaculum—radiocarpal capsule	Acute pain mimicking DeQuervain disease 15 days before. Mild improvement with NSAID	Mixed. Mostly Type III, in part II	Yes	Yes	No		Phone call 1 month. Improved
18	M	41	9 mm	Dorsal capsule CMCj (Capitate‐3rd MC)	Atraumatic swelling and pain dorsal wrist 1 week before, improved at time of US exam	Type III	No	Yes	No	Intraosseus migration	No
19	M	56	12 mm	Dorsal capsule CMCj (Capitate‐2nd‐3rd MC)	Acute dorsal CMCj pain.	Type II	Yes	Yes	No		No
20	F	55	11 mm	Capsule triscaphe joint	Wrist pain	Type III	Yes	Yes	X‐ray, CT		No
21	F	46	10 mm	Dorsal intercarpal capsule (lunate‐hamate)	Acute dorsal CMCj pain	Type I	No	No	X‐ray	Glomic tumor 5th finger	Improved in 1 month (time from X‐ray and US)
22	F	66	11.5 mm	Ecrl insertion (CMCj)	Acute dorsal CMCj pain.	Type III	Yes	No	No		US. Improvement 3 week
23	F	80	6 mm	Ecrb insertion (CMCj)	Chronic dorsal wrist pain, recently exacerbated	Type III	No	No	No	CMCj oa	No
24	F	17	6.5 mm	Radiolunotriquetral ligament (triquetrum)	Dorsal wrist pain 1 month.	Type III	No	Yes	No		No
25	F	37	6 mm	Dorsal intercarpal ligament/capsule (triquetrum)	Acute dorsal wrist pain 1 month before. Already improved with rest/NSAID	Type III	No	No	No	Intraosseus migration	Phone call 1 month. Resolved
26	F	17	5 mm	Dorsal intercarpal ligament/capsule (scaphoid)	Chronic dorsal wrist pain, increased in the last month	Type III	No	No	No		US at 4 months. Similar image, improvement with rest; although pain represented with sport activities
27	M	61	15 mm	1st MC Head. Opponens muscle	Acute base thumb pain 5 days before	Type III	Yes	Yes	No	Intramuscular migration	US. Improvement 3 week
28	F	29	7.5 mm	2nd MCPj—RCL	Acute pain MCPj exacerbated by maximal ROM	Type III	No	Yes	X‐ray		No
29	F	49	6 mm	2nd MCPj—lumbrical	Acute pain II MCPj radial side 2 weeks before. Already mild improvement	Type III	No	Yes	No		US. Improvement 3 week
30	F	41	7 mm	Peritendinous FDS‐FDP, MCPj 4th finger	Finger pain	Type III	Yes	Yes	X‐ray (with US); MRI (2 months later)		Pain improved in 3 weeks, completely disappeared at MRI 2 months later with resolution
31	F	49	5 mm	Capsule/sagittal band MCPj 5th finger	Finger pain	Type II	Yes	Yes	X‐ray		No
32	F	66	4 mm	Interossei/RCL 4th MCPj	Chronic pain base 4th finger. 1 month before acute episode pain. Slight improvement at US examination	Type III	Yes	Yes	X‐ray (6 month and 1 month before US)		Resolved 2 months after US
33	F	54	2.5 mm	Lumbricals/interossei 5 h MCPj	Persistent atraumatic pain	Type III	Yes	Yes	X‐ray		No
34	M	47	5 mm	A2 pulley distal insertion 3rd finger	Painful flexion of 3rd finger	Type I	No	Yes	X‐ray	Bone callus at the insertion of PIPj volar plate (previous fracture)	Stable after 2 months from X‐ray
35	F	54	10 mm	2nd PIPj—UCL	Acute finger pain 1 week before. No improvement with oral steroid	Type III	Yes	Yes	No		US. Resolution 6 months
36	F	54	3 mm	2nd PIPj—UCL	Persisting pain after minor trauma 2nd finger	Type III	No	Yes	X‐ray		No
37	F	29	7 mm	Dorsal capsule PIPj 5th, deep to central band	Persisting pain after 5th finger dislocation (4 weeks after trauma)	Type I	No	No	X‐ray	Stretch injury of RCL; volar plate PIPj partial tear	No

Abbreviations: Calc. Type indicates the type of the calcification; CMCj, MCPj, PIPj, PTj indicates respectively the carpometacarpal, metacarpophalangeal, proximal interphalangeal, pisotriquetal joint; CTS, carpal tunnel syndrome; Edem. Ch., edematous changes of the adipose soft tissue contiguous to the calcification; FCU, flexor carpi ulnaris; FDS and FDP, flexor digitorum superficialis and profundus; OA, osteoarthritis; RCL and UCL, radial and ulnar collateral ligament; ROM, range of movement; UN, ulnar nerve.

**FIGURE 1:**
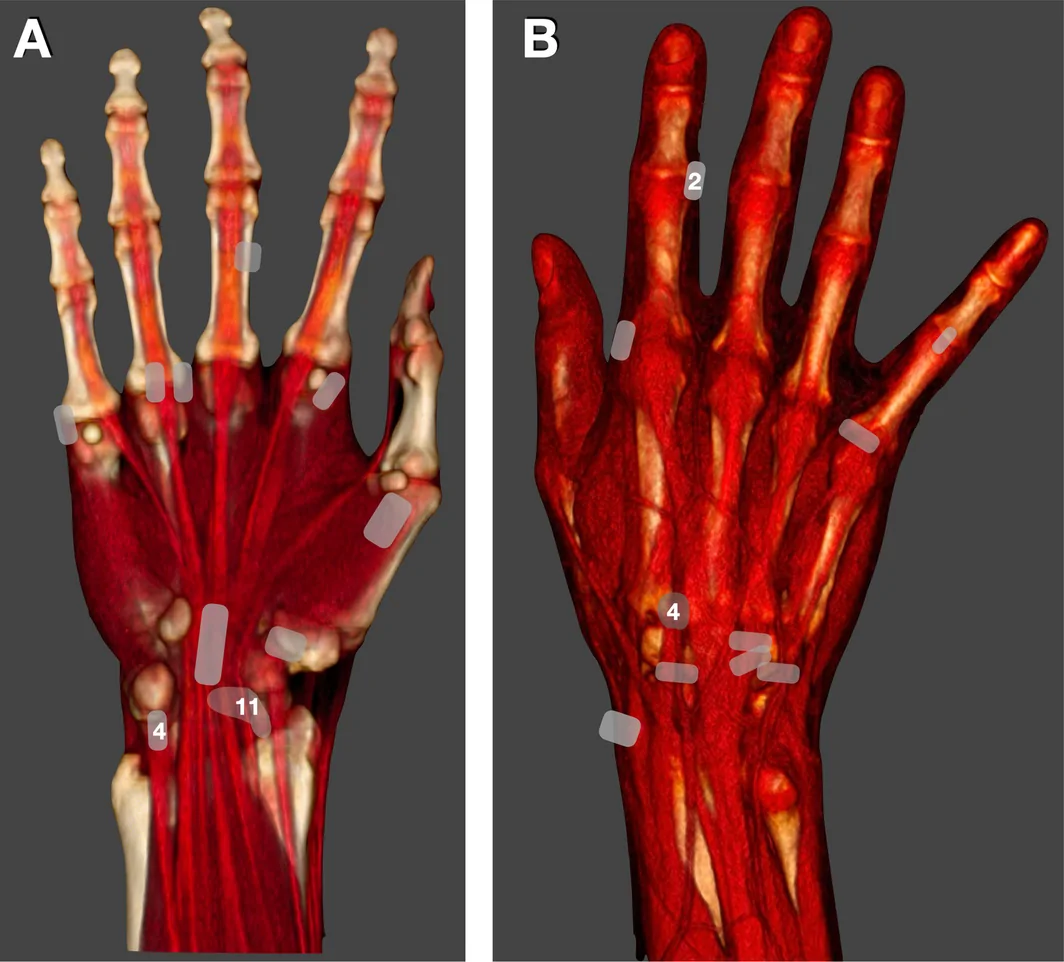
CT‐3d based schematic drawings (A, volar; B, dorsal) showing the location of the calcific deposit in the present case series. The number indicates similar cases. Deposits that were found mostly at collateral ligaments are shown in the dorsal image, as well as the deposit of the 1st extensor compartment. Calcium deposits located mostly along the intrinsic muscle/tendon are shown in the volar image.

Patients were mostly women (30/37). The mean age was 50 years (ranging from 17 to 80 years).

The mean size of the calcification was 11.2 mm (ranging from 2.5 to 47 mm).

Calcifications were mostly (73% of the cases) in the resorptive phase (colliquated, Type III); in addition, three cases showed mixed calcification, in part Type III (Table 2). All of the patients presented with pain, mostly non‐traumatic. In patients with recent trauma, adjunctive imaging techniques (radiography or CT) were used to exclude fractures or bony avulsion injuries and avoid misdiagnosis.

**TABLE 2:** Percentage of frequency of calcific deposits found at different anatomical locations.

Anatomical location	Frequency	Percentage
Volar wrist. Flexor tendons	1	2.7%
Volar wrist. Ligament‐Capsule	11	29.7%
FCU	4	10.8%
1st compartment retinaculum—radiocarpal capsule	1	2.7%
Dorsal capsule CMCj	2	5.4%
Capsule triscaphe joint	1	2.7%
Dorsal intercarpal capsule/ligament	3	8.1%
Ecrl/ecrb insertion (CMCj)	2	5.4%
Radiolunotriquetral ligament (triquetrum)	1	2.7%
1st MC Head. Opponens muscle	1	2.7%
Around MCPj (CL, lumbricals, interossei, flexor tendons, joint capsule)	6	16.2%
A2 pulley distal insertion 3rd finger	1	2.7%
PIPj	3	8.1%

Abbreviations: CL: collateral ligament; CMCj, MCPj, PIPj: indicate the carpometacarpal, metacarpophalangeal, proximal interphalangeal joint; FCU: flexor carpi ulnaris.

Edematous changes in the neighboring tissue were present in 51% of patients, of which 17/19 showed type III calcification, whereas hyperemia was present in 69% of the cases, of which 24/29 presented type III calcific deposits. X‐ray was available in 13 patients, magnetic resonance (MRI) in 3, and computed tomography (CT) in 2.

Follow‐up clinical data were available for 16 out of 37 patients, among whom 6 underwent another US examination. These follow‐up data were obtained mostly about 1 month after the first US exam. Complete resolution of symptoms was observed in four patients. Gradual, partial improvement was seen in 11 patients. One patient, the one with a previous fracture, at 2 months reported similar symptoms. One patient reported a sudden improvement after a local US‐guided injection (steroid and local anesthetic), although we have no other follow‐up information.

## 4 Discussion

US is a well‐known imaging modality to diagnose rotator cuff HADD; however, it is poorly reported in the literature for the diagnosis of HADD of the hand/wrist. All of our patients referred pain, mostly non‐traumatic.

The largest series, to the best of our knowledge, is the one presented by Carroll (Carroll et al. 1955). In 100 acute HADD of the hand diagnosed by conventional radiography, he found 37 cases at the FCU tendon, near the pisiform bone, which was the most common site. Other commonly reported locations included the ligaments of the fingers and the short intrinsic musculature in the palm.

We also found the flexor carpi ulnaris (FCU) tendon to be involved in four cases (Figure 2). Ultrasound was also useful in demonstrating concomitant pisiform bursitis in two cases. This involves an inconstant bursa located deep to the FCU tendon, which can be visualized on ultrasound when distended (Figure 2C) (Draghi et al. 2014). One patient presented with dysesthesia related to the proximity of the calcification to the ulnar nerve.

**FIGURE 2:**
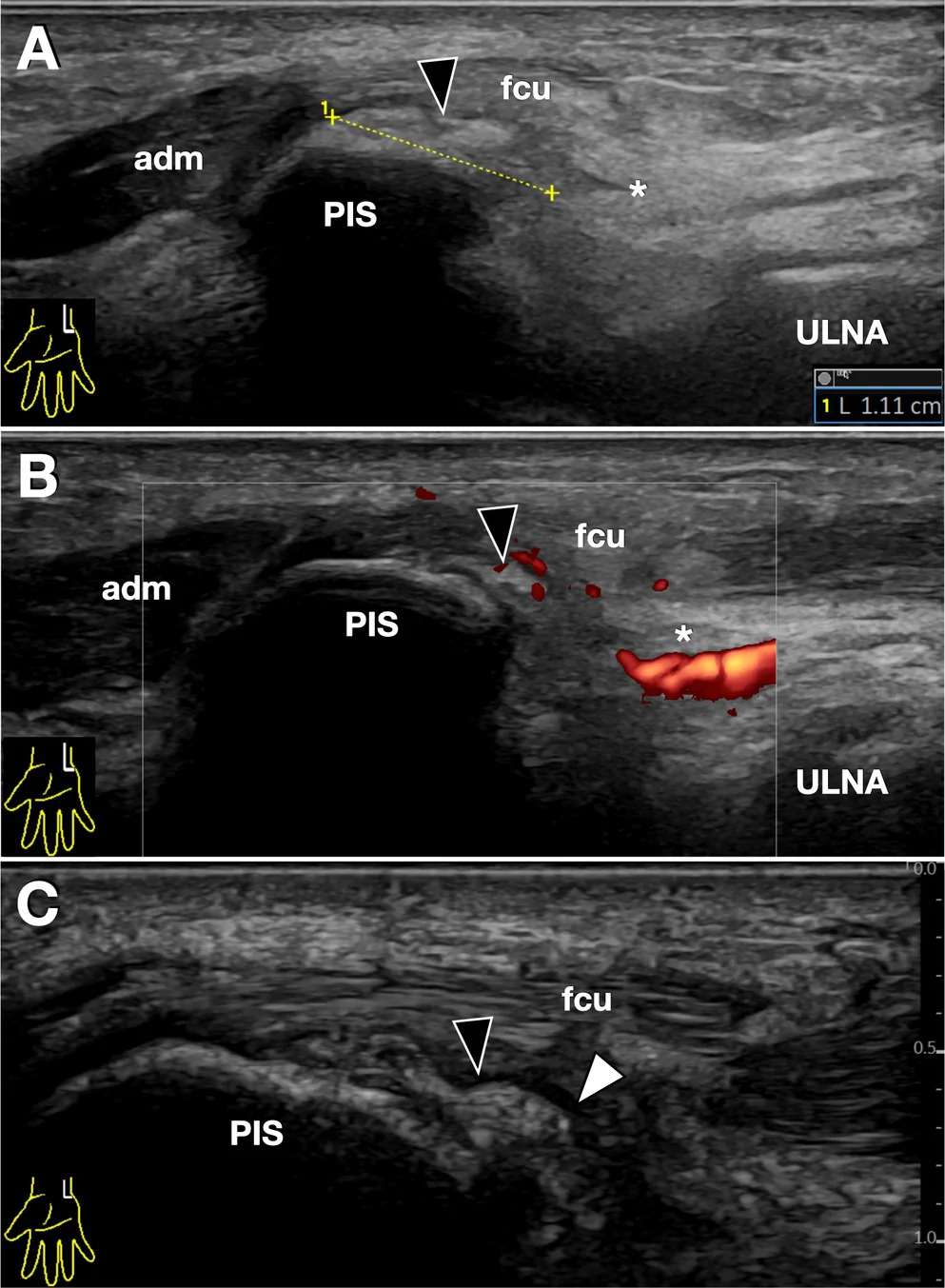
Patient 13. A 30‐year‐old male with FCU HADD. (A, B) Sagittal b‐mode (A) and power Doppler (B) sonograms with a 16 MHz transducer along the long axis of the flexor carpi ulnaris (fcu). This 30‐years‐old male presented with acute pain of the ulnar side of the wrist, local redness. US was performed 5 days after the acute onset of symptoms. The patient reported a slight improvement with NSAID. A centimetric calcific deposit (black arrowhead) can be depicted at the distal part of the FCU tendon, with contiguous edematous changes (asterisk), and hyperemia. Pressure with the probe elicited the patient's pain. (C) Corresponding hockey stick image (20 MHz) demonstrates the calcific deposit mostly migrated to the pisiform bursa (white arrowhead). Pain almost resolved at phone follow‐up 1 month later. Adm indicates the abductor digiti minimi muscle; PIS, pisiform bone.

A common location in our series (11/37 cases) is the ventral aspect of the wrist capsule/ligaments (Figure 3, Movie 1); a possible explanation for the difference with Carroll's series is the ability of US to better depict small calcifications at this location with respect to conventional radiography (due to the superposition of bony structures, in particular if a lateral view is not obtained). In fact, deposits near the carpal bones tend to be more difficult to identify, and a small deposit may be challenging to detect (Nikci and Doumas 2015; Diekhoff and Ulas 2025).

**FIGURE 3:**
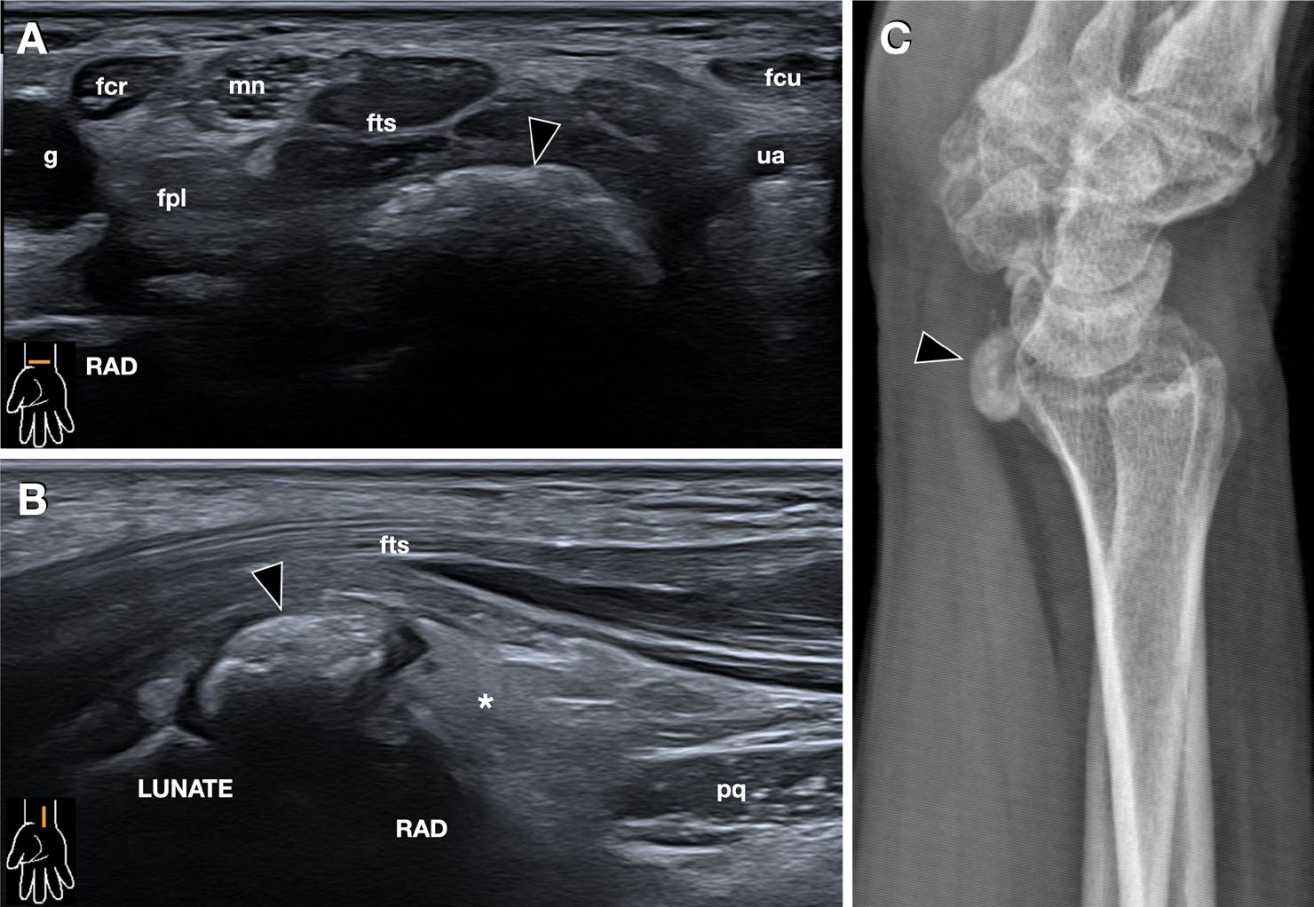
Patient 11. Volar Wrist capsule HADD (A, B) Axial (A) and sagittal (B) sonograms obtained at the level of the wrist in a 59‐year‐old patient presenting with volar wrist acute pain. Note the cloudlike colliquated calcific deposit (black arrowhead) deep to the flexor tendons (fts) and the contiguous edematous changes. Flexion and extension of the fingers confirmed the deposit was non‐intratendinous, but located in the wrist volar capsule/ligament. Of interest, note in (A) a ganglion cyst (g) of the volar radio‐carpal joint. (C) Lateral X‐ray demonstrates the calcific deposits (black arrowhead) along the volar side of the wrist. fcr indicates the flexor carpi radialis; fcu, flexor carpi ulnaris; fpl, flexor pollicis longus; fts, flexor tendons; mn, median nerve; pq, pronator quadratus; RAD, radius bone; ua, ulnar artery.

Volar wrist HADD may also present with carpal tunnel syndrome (CTS), in 9 of the 12 patients (including calcific deposits at capsule, ligaments or tendons). Thisassociation that we have found suggests that, in the case of acute CTS symptoms and wrist pain, HADD should be considered as a possible differential diagnosis. The median nerve crosses the carpal tunnel, which is a narrow osteofibrous tunnel; the calcific deposit may compress/dislocate the nerve or cause inflammation of the synovial recess/tendon sheath, leading to nerve irritation (Diekhoff and Ulas 2025; Becciolini et al. 2025).

We found four cases of calcific deposit near the dorsal carpometacarpal joint (CMCj), possibly mimicking clinically a carpal boss, which is related to bony abnormalities at the joint between the capitate and the 2nd‐3rd metacarpal, at the quadrangular joint of the wrist. In congenital carpal boss, US can depict a small ossicle, whereas in the acquired type, it can show local osteophytes and concomitant soft tissue pathology (Corvino et al. 2024).

Intraosseous migration of HADD is an infrequent complication, mostly known at the shoulder, in which cortical erosion may appear, related to calcium migration. US can demonstrate it by showing a bone focal discontinuity confirmed in two orthogonal sonograms, near a calcific deposit, without a traumatic fracture (Bianchi and Becciolini 2019; Malghem et al. 2015; Della Valle et al. 2015) Intraosseous migration has also been recently described in the hand/wrist (Goller et al. 2021) In our series, we could demonstrate intraosseous involvement in 4 patients (Figure 4).

**FIGURE 4:**
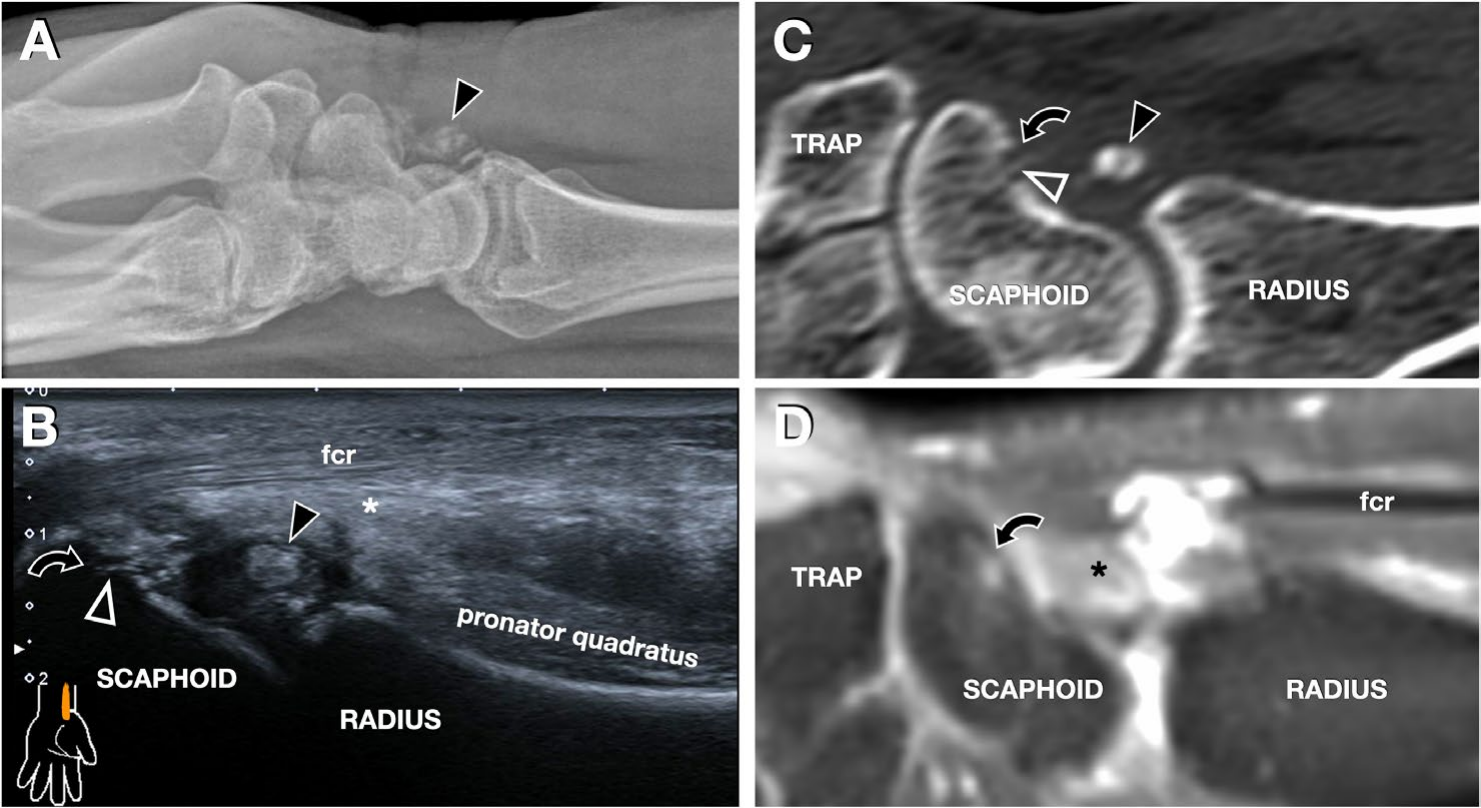
Patient 2. Volar wrist HADD; intraosseous migration. Imaging correlation. (A) Conventional radiography obtained 3 days after the acute onset of atraumatic volar wrist pain, in a 45‐year‐old woman, demonstrates calcific deposits along the volar side of the wrist. (B) Longitudinal sonogram at the volar radiocarpal joint (radio‐scaphoid) obtained the same day. Note the calcific deposit along the wrist capsule (black arrowhead). Anteriorly, a second deposit is near the scaphoid, which shows a discontinuity (curved arrow) where the calcium migrated intraosseous (open arrowhead). There are edematous changes of the neighboring tissue (asterisk), appearing diffusely hyperechoic. (C) Corresponding sagittal CT obtained 10 days later confirms the calcific deposit (black arrowhead) as well as the mild bony irregularity related to the intraosseous migration. (D) MRI obtained 1 week after the CT study. The deposit is difficult to demonstrate. Note the edema (asterisk) in the region where US and CT show the calcium deposit, near the flexor carpi radialis (fcr, partially shown) and the small subcortical erosion on the scaphoid distal pole (curved arrow) with associated mild bone marrow edema. Rest and NSAIDs were suggested with progressive clinical improvement at one and 3 months. A final diagnosis of acute periarticular inflammation due to HADD of the volar wrist capsule, with concomitant CTS was proposed.

At the palm and near the metacarpophalangeal joint (MCPj), intrinsic hand muscles were involved in 4 patients. In one patient, the deposit showed an intramuscular migration (Figure 5). This complication is rare in calcific tendinopathy but is reported in particular at the shoulder (Becciolini et al. 2016; Pereira et al. 2016).

**FIGURE 5:**
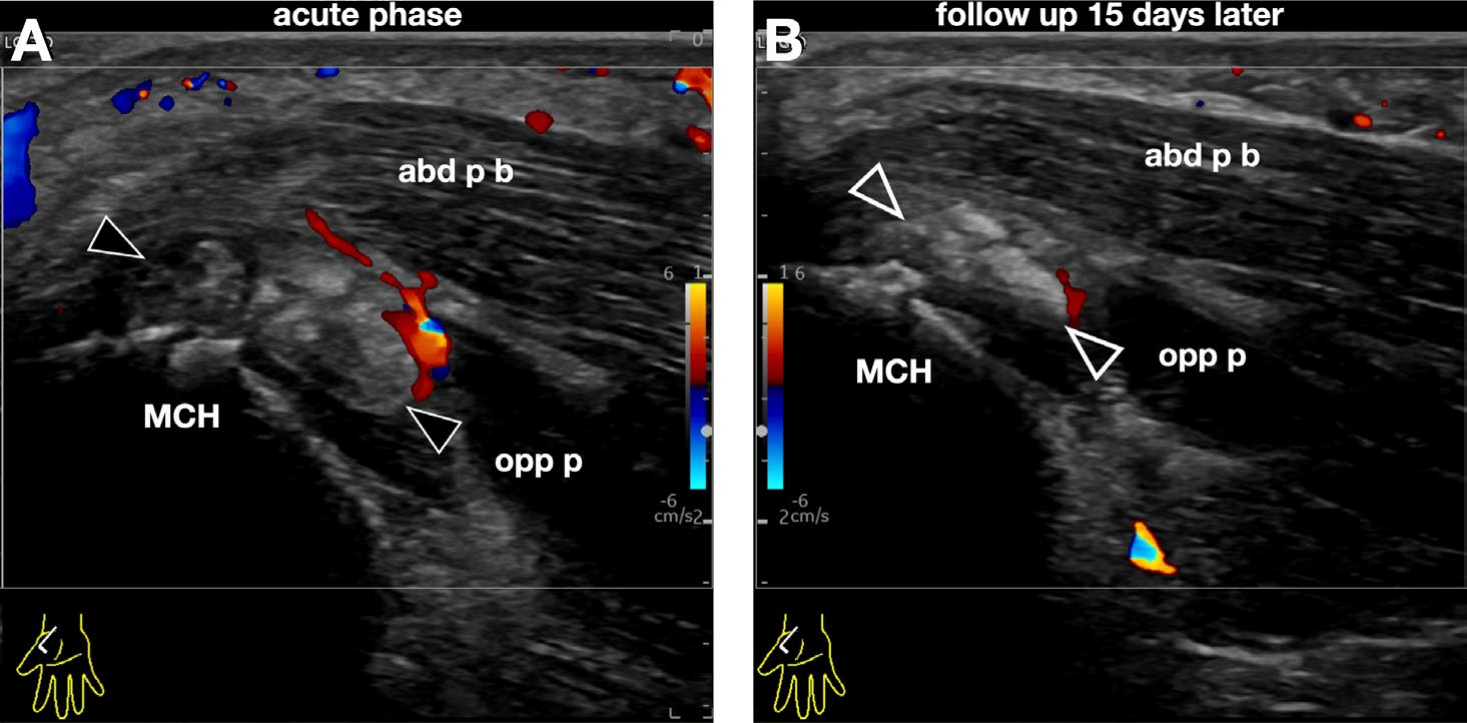
Patient 27. Intramuscular HADD migration in a 60‐year‐old man. Interest of the US follow‐up. (A, B) color Doppler sonograms obtained in the same patient, in the acute phase of pain at the base of the thumb (A), and 15 days later (B). The US machine has a function (“Compare Assistant”) which allows for comparative images with the same settings (depth, frequency, and Doppler settings), to ensure a better understanding of modification during follow‐up. Note in (A) the amorphous deposit (black arrowheads) inside the opponens pollicis muscle (opp p), with edematous and hyperemic changes also extending to the abductor pollicis brevis (abd p b) and subcutaneous fat. In (B), 15 days later, symptoms improved. The calcific deposit is smaller (open arrowhead) and also the edematous changes as well as hyperemia decreased. MCH indicates the 1st metacarpal head.

Finger deposits were found at the collateral ligaments (Figure 6A,B, Movie 2), A2 pulley, articular capsule, or near the flexor tendons. One calcific deposit near the MCPj was misinterpreted on X‐ray for a normal sesamoid (Figure 6C). Review of the radiograph following the ultrasound examination enabled differentiation of the morphology of HADD from that of a normal sesamoid bone, the latter being characterized by a denser cortical outline and a preserved internal trabecular architecture.

**FIGURE 6:**
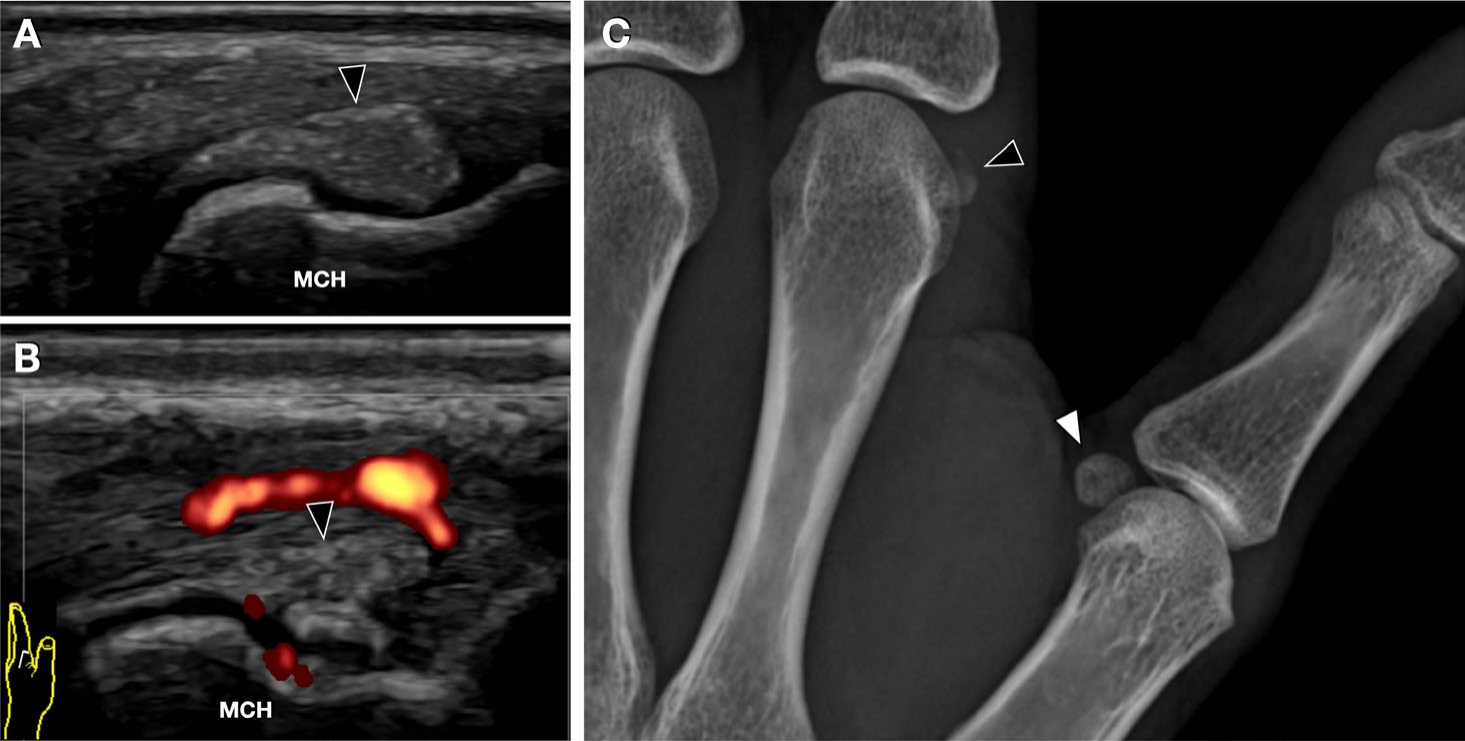
Patient 28. Radial Collateral ligament (RCL) HADD in a 29‐year‐old woman. X‐Ray pitfall. (A, B), Coronal b‐mode (A) and power Doppler (B) sonograms in a patient with acute symptoms at the radial aspect of the 2nd MCPj, in the absence of trauma. Acute pain was present with maximal ROM movements, especially hyperextending the joint. Note an amorphous hyperechoic deposit (black arrowhead) in the region of the RCL, without acoustic shadowing, and contiguous hyperemia. Pressure was painful. (C) Corresponding X‐ray, which was interpreted as normal. Note the different bony structure of a normal sesamoid (white arrowhead) of the thumb, versus the morphology of the HADD deposit at the 2nd MCPj (black arrowhead), without a cortical or trabecular appearance. MCH indicates the 2nd metacarpal head.

For the 16 patients with known follow‐up, we confirm that HADD is mainly a self‐limiting condition, with almost every patient except one showing at least a partial resolution of symptoms over time.

US‐barbotage may also be employed to reduce symptoms; in particular, in a recent review of 41 patients with HADD outside the shoulder, it was used at the wrist for the extensor carpi radialis longus (Albano et al. 2024). In one of our patients, a US‐guided injection (steroid and local anesthetic) was proposed, with improvement of the symptoms after it.

The US findings in HADD differ in the function of the different stages. Moreover, the acoustic attenuation of the calcification also depends on the crystal concentration and the size of the deposit (Tamborrini et al. 2023). In the formative and resting phase, the deposit usually appears well‐defined, hyperechoic with a complete (Type I) or faint acoustic shadowing (Type II). In the acute resorptive stages, usually the calcific deposit fragments and appears ill‐defined, cloudlike, lobular, without acoustic shadowing (Type III). During this phase the calcium crystals may migrate into the neighboring structures, with inflammation and edematous changes in the involved tissue and hyperemia at Doppler, as present in many cases of our series (Bianchi and Becciolini 2019; Krolikowski et al. 2022). Most of our patients (26/37) presented with Type III calcific deposits, supporting the evidence that HADD is typically symptomatic during the resorptive phase, when patients are more likely to seek medical attention because of severe pain.

Regarding other imaging modalities, X‐ray is the usual reported method to diagnose hand/wrist HADD, being cost‐effective (Siegal et al. 2009). However, in the series by Carroll, only 73/100 radiographs were correctly interpreted at first (Carroll et al. 1955; Nikci and Doumas 2015). X‐ray is particularly valuable when prior radiographic imaging performed before symptom onset is available for comparison (Figure 7). Advanced imaging modalities (CT, MRI), usually not needed, may be considered in patients with acute, persisting symptoms (e.g., intraosseous migration of the deposit) or when a surgical approach is hypothesized. CT remains the best modality to describe the consistency of the calcific deposit and may be useful in case of osseous involvement, but it is limited by radiation, accessibility, and costs (Siegal et al. 2009). MRI has limitations in clearly depicting small calcification, possibly giving misleading results; however, associated with other modalities, it can be useful, being the most appropriate to demonstrate bone marrow edema in intraosseous migration (Figure 4D) (Bianchi and Becciolini 2019; Malghem et al. 2015).

**FIGURE 7:**
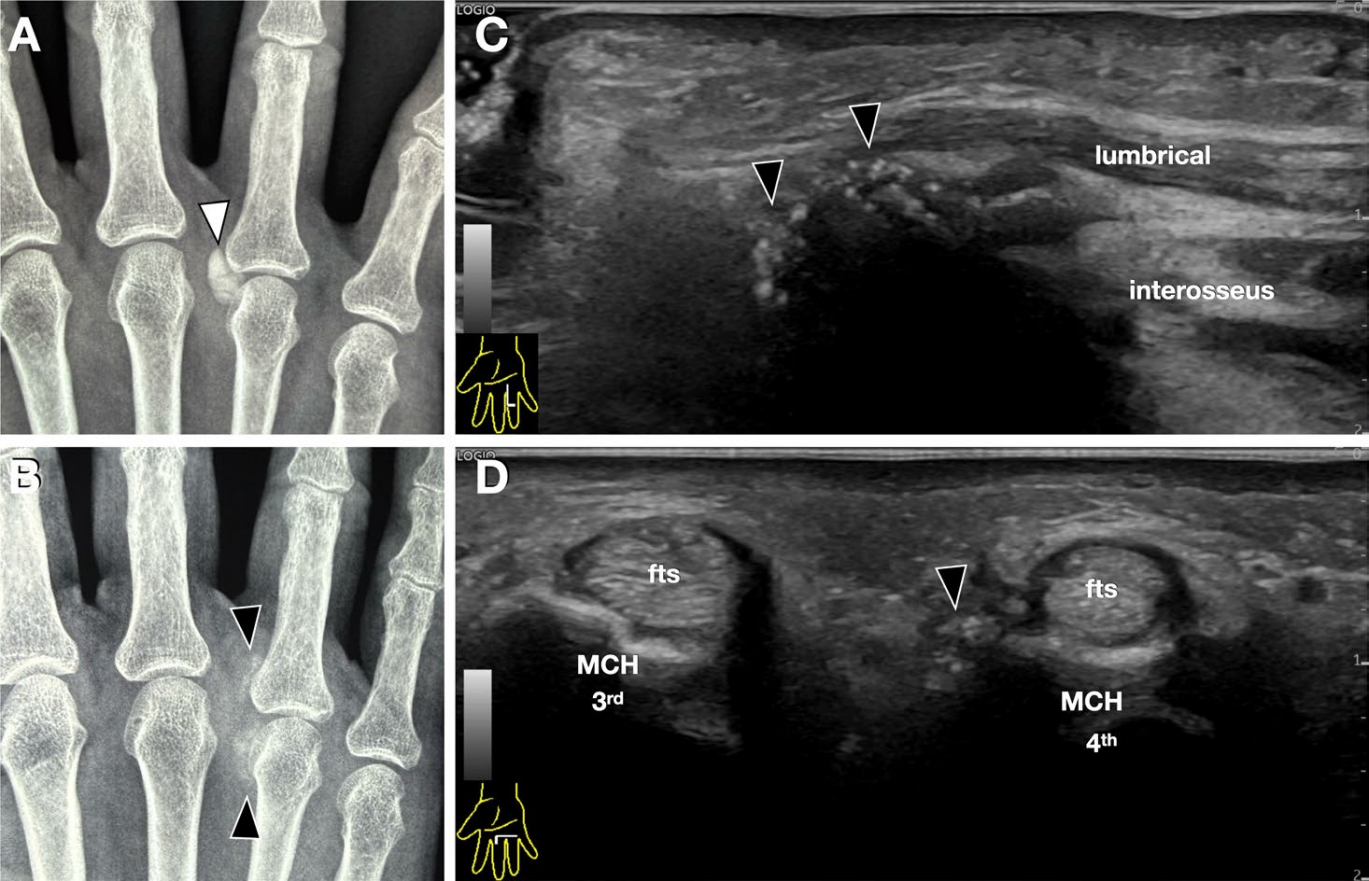
Patient 32. Interosseous/RCL 4th MCPj HADD in a 66‐year‐old woman. Interest of comparison X‐Ray (A, B). Conventional radiography obtained 6 months before (A) and at the time of an acute HADD resorptive attack (B). Note in (A), a well‐defined calcific density (white arrowhead) at the radial aspect of the 4th MCPj. At the time of the first radiograph, the patient reported discomfort in that region. In (B), note the change in the calcification morphology, now fragmented and hazy, amorphous, corresponding to the resorptive phase. (C, D) Sagittal (C) and axial (D) sonograms obtained 3 weeks after the second X‐ray, still depict an amorphous deposit along the interosseous tendon and at the RCL of the MCPj. Symptoms were already improving, and resolved 2 months later, as reported by the patient. MCH indicates the metacarpal head (respectively of the third and fourth fingers).

The possible main differential diagnosis in crystal disease of the hand includes CPPD and gout (Omoumi et al. 2016; Diekhoff and Ulas 2025). CPPD is related to calcium pyrophosphate dihydrate deposit and is the most prevalent form at the hand, particularly in the elderly (Diekhoff and Ulas 2025). Metabolic diseases, such as hemochromatosis, are associated. Typical locations include the triangular fibrocartilage, intrinsic and extrinsic ligaments of the wrist. Acute inflammation may give rise to the so‐called pseudogout attack. The hyperechoic deposits don't show acoustic shadowing, unlike HADD (Type I and II). Moreover, CPPD deposits usually appear linear, punctate, or granular (Goller et al. 2021). In advanced stages, at the wrist, it is not uncommon to depict diffuse hyperechoic granular images along the capsule (Figure 8). A hallmark of CPPD, which helps in the diagnosis, is the presence of a hyperechoic deposit in the middle layer of cartilage.

**FIGURE 8:**
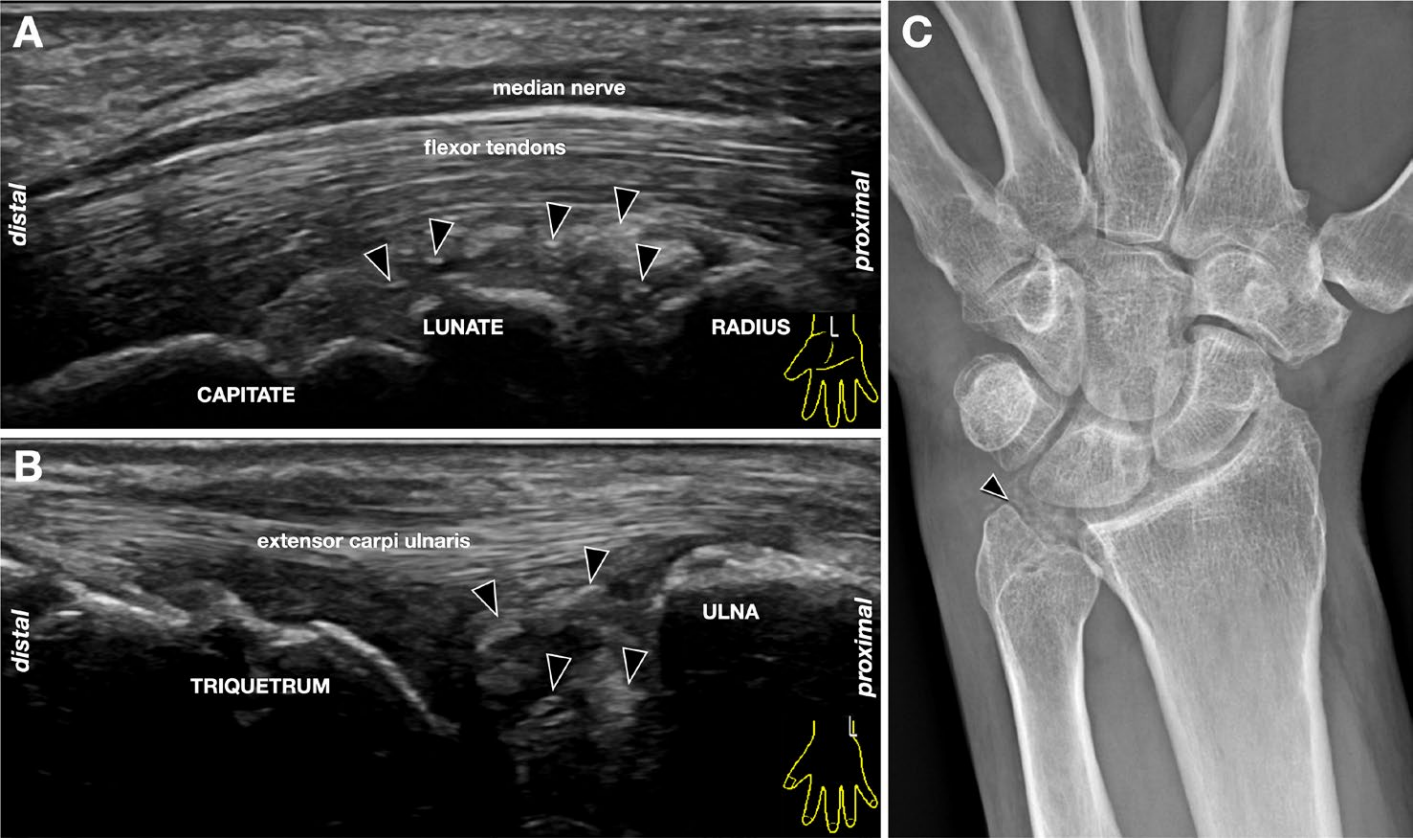
Differential diagnosis. Wrist chondrocalcinosis. (A, B) Sagittal volar (A) and ulnar coronal (B) sonograms, with wrist X‐ray (B) in a 70‐year‐old female patient with CTS and episodes of acute wrist pain. In (A), B‐mode sonogram shows bulging of the volar capsule and ligaments of the wrist due to diffuse calcific deposits (black arrowheads). Note the appearance of the deposits: Diffuse, linear, punctate (black arrowheads). In (B), the same deposits can be found at the level of the TFCC. (C) the X‐ray image shows calcific deposits within the TFCC (open arrowheads), typical of chondrocalcinosis.

Gout is related to monosodium urate deposition in patients with hyperuricemia. Gout deposits may be heterogeneous, hyperechoic aggregates or tophi, with various echostructures and possibly acoustic shadowing and a hypoechoic halo. The presence of a double contour hyperechoic sign of the cartilage, extra‐articular erosions, may help the diagnosis, associated with clinical and laboratory findings (Figure 9). Given the characteristic imaging findings associated with different crystal arthropathies, CPPD and gout were excluded. Furthermore, all cases of HADD were independently reviewed by the US examiners, and patients with a history of gout attacks or elevated serum uric acid levels were excluded.

**FIGURE 9:**
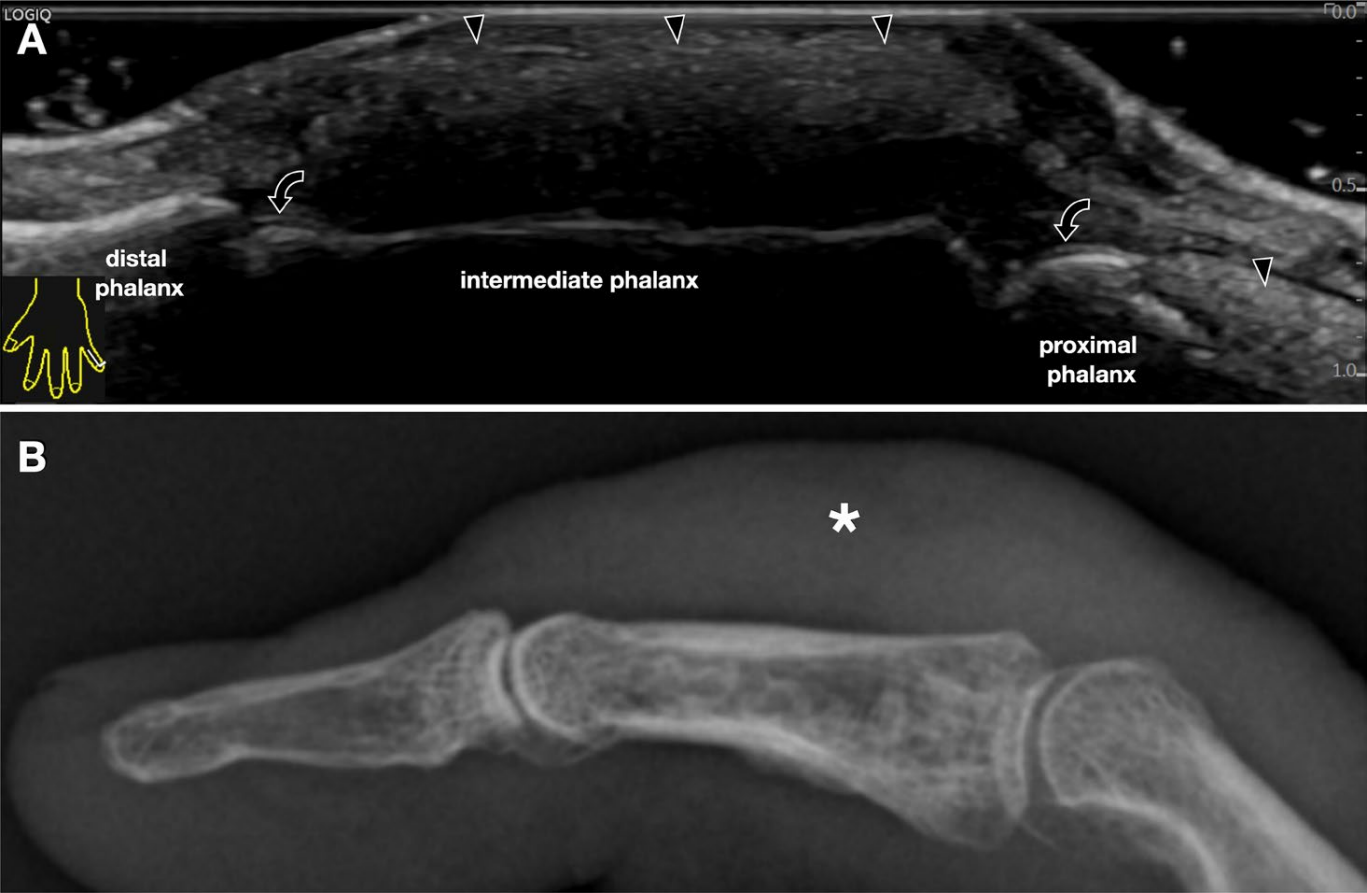
Differential diagnosis. Finger gout. (A, B) Sagittal sonogram (A) and X‐ray (B) of the 5th finger in a 55‐year‐old male patient with known gout, high serum uric acid level, and a mass. In (A), ultrasound shows hyperechoic diffuse aggregates (black arrowheads) with partial acoustic shadowing, in the subcutaneous tissue of the intermediate phalanx, over the lateral bands of the extensor tendon (not appreciable due to the aforementioned acoustic shadow). A deposit is also located over the proximal phalanx, under the central band of the extensor. Note the hyperechoic double contour lines at the proximal and distal dorsal interphalangeal joint cartilage (curved arrows). In (B), the X‐ray image shows an amorphous soft‐tissue mass (asterisk) without a clear calcific deposit. The clinical and imaging features were suggestive of gout deposits. The patient underwent surgery for similar, larger deposits, and gout was confirmed upon analysis.

## 5 Limitations

The main limitation of our work is the lack of analysis of the calcific deposit to precisely characterize its nature.

Another limitation of our study is the lack of correlation with other imaging modalities for many of the patients.

A further limitation of our study is that we lack data on the prevalence of this pathology.

## 6 Conclusions

US can be a precise imaging modality to demonstrate calcific deposits in the hand and wrist. The peculiar findings suggest the diagnosis of HADD. Follow‐up (e.g., phone call) is suggested to demonstrate the benign evolution of the pathology. In most of our cases, the diagnosis was straightforward, and patients were informed that the condition is typically self‐limiting; therefore, further imaging was often unnecessary (see for example case 27, Figure 5). When additional imaging was required to confirm the diagnosis, plain radiography was the most commonly requested modality, as it can demonstrate calcific deposits with a substantially lower radiation burden than CT.

## Funding

The authors have nothing to report.

## Disclosure

We confirm that this work is original and has not been published elsewhere nor is it currently under consideration for publication elsewhere. Publication is approved by all authors. Each author participated sufficiently in any submission to take public responsibility for its content.

## Ethics Statement

The research was conducted according to the guidelines of the Declaration of Helsinki. Authors declare that all the procedures being performed were part of the routine care, all the collected data were anonymized, and no information is linked or linkable to a specific person.

## Consent

The requirement for informed consent was waived due to the retrospective design and anonymized data.

## Conflicts of Interest

The authors declare no conflicts of interest.

## Supporting information


**Movie 1:** Patient 11. Volar Wrist capsule HADD. Same as Figure 3
(A, B) Axial (A) and sagittal (B) live sonograms obtained at the level of the wrist in a 59‐year‐old patient presenting with volar wrist acute pain. Note the cloudlike colliquated calcific deposit (black arrowhead) deep to the flexor tendons (fts) and the contiguous edematous changes. Flexion and extension of the fingers confirmed the deposit was non‐intratendinous but located in the wrist volar capsule/ligament. Of interest, note in (A) a ganglion cyst (g) of the volar radio‐carpal joint.
(C) Lateral X‐ray demonstrates the calcific deposits (black arrowhead) along the volar side of the wrist.
fcr indicates the flexor carpi radialis; fcu, flexor carpi ulnaris; fpl, flexor pollicis longus; fts, flexor tendons; mn, median nerve; pq, pronator quadratus; RAD, radius bone; ua, ulnar artery.

**Movie 2:** Patient 28. Radial Collateral ligament (RCL) HADD in a 29‐year‐old woman. X‐Ray pitfall. Same as Figure 6
(A, B), Coronal b‐mode (A) and power Doppler (B) sonograms in a patient with acute symptoms at the radial aspect of the 2nd MCPj, in the absence of trauma. Acute pain was present with maximal ROM movements, especially hyperextending the joint.
Note an amorphous hyperechoic deposit (black arrowhead) in the region of the RCL, without acoustic shadowing, and contiguous hyperemia. Pressure was painful.
The deposit is better depicted in (B), with a higher frequency hockey stick transducer (L6‐24). A is obtained with a ML4‐20 transducer.
(C) Corresponding X‐ray, which was interpreted as normal. Note the different bony structure of a normal sesamoid (white arrowhead) of the thumb, versus the morphology of the HADD deposit at the 2nd MCPj (black arrowhead), without a cortical or trabecular appearance.
MCH indicates the 2nd metacarpal head.

## Data Availability

The data that support the findings of this report are available on request from the first author (Marco Becciolini). The data are not publicly available due to restrictions that could compromise the privacy of patients.
